# Implementing adaptive strategies for infection prevention and control in resource-limited settings

**DOI:** 10.3389/fmed.2026.1883213

**Published:** 2026-07-07

**Authors:** Ammar Ali Al-Raimi

**Affiliations:** Department of Health Management, College of Business Administration, Al-Razi University, Sana’a, Yemen

**Keywords:** hand hygiene adherence, healthcare worker compliance, healthcare-associated infections, hospital-acquired infections, infection prevention and control, patient safety, personal protective equipment

## Abstract

**Background:**

Healthcare-associated infections (HAIs) are a major challenge in resource-limited hospitals, where conventional infection prevention and control (IPC) protocols are difficult to implement because of infrastructure constraints, limited personal protective equipment (PPE), and understaffing. Adaptive IPC strategies that are tailored to local conditions may improve patient safety and compliance. This study aimed to evaluate the effectiveness, feasibility, and sustainability of adaptive strategies for reducing hospital-acquired infections and improving protocol adherence in a resource-limited hospital setting.

**Methods:**

A quasiexperimental mixed-methods study was conducted over 12 months in multiple inpatient wards of “A” Hospital in Yemen, with assessments at baseline, 3 months, 6 months, and 12 months. The interventions included locally optimized equipment disinfection protocols, low-cost staff training, peer mentorship, visual reminders, and spatial workflow reorganization. Intervention outcomes were compared with historical control data from comparable wards via a difference-in-differences approach. Hospital-acquired infection outcomes were analyzed via Poisson or negative binomial regression with patient days as the exposure offset, whereas adherence outcomes were analyzed via mixed-effects logistic regression.

**Results:**

The final analytic dataset included 2,420 admissions contributing 13,980 patient-days. The cumulative incidence of hospital-acquired infection decreased from 15.3% at baseline to 8.2% at 12 months, corresponding to an absolute reduction of 7.1 percentage points and a relative reduction of 46.4%. In the adjusted negative binomial regression using patient days as the exposure offset, the 12-months HAI rate was significantly lower than that at baseline (adjusted IRR 0.52). Difference-in-differences analysis revealed an additional 6.2-percentage-point reduction in cumulative HAI incidence compared with historical controls. Adherence to hand hygiene, PPE use, and equipment disinfection increased to 83.0%, 80.1%, and 76.1%, respectively. Resource utilization increased during the intervention period.

**Conclusion:**

Adaptive, context-specific infection prevention and control strategies were associated with significant reductions in hospital-acquired infection rates over a 12-months period and improved healthcare worker adherence in a resource-limited hospital setting. The findings suggest that low-cost, locally adapted interventions can be integrated into routine clinical workflows and implemented with manageable increases in resource use. This study provides a practical framework for implementing and evaluating adaptive infection prevention and control interventions in similar low-resource healthcare environments.

## 1 Introduction

Healthcare-associated infections are a major global public health and patient safety challenge (1). They contribute substantially to preventable morbidity, mortality, prolonged hospitalization, antimicrobial use, and increased healthcare costs (2). Although healthcare-associated infections affect healthcare systems worldwide, their burden is especially pronounced in resource-limited settings, where hospitals often face persistent structural and operational barriers to infection prevention and control (3). These barriers include inadequate infrastructure, limited sterilization capacity, shortages of personal protective equipment, insufficient infection control staffing, overcrowding, and inconsistent availability of essential supplies (3, 4). As a result, hospitals in resource-constrained environments may experience higher infection rates and lower adherence to recommended infection prevention and control practices than hospitals in better-resourced settings (3). Conventional infection prevention and control programs are often designed for healthcare systems with stable supply chains, adequate staffing, dedicated infection control teams, advanced sterilization technologies, and reliable access to disposable equipment (5, 6). However, these assumptions do not always apply in low-resource hospitals, where staff may be required to deliver care under conditions of supply instability, high patient volume, limited space, and competing clinical priorities (3). Therefore, directly transferring infection control models from high-income settings to resource-limited hospitals may be impractical or unsustainable unless they are adapted to the local context. Adaptive infection prevention and control strategies offer a practical alternative by modifying implementation approaches according to local resources, workflows, and constraints (7). Such strategies may include optimizing the use of locally available disinfectants, standardizing equipment disinfection procedures, using short low-cost staff training sessions, introducing visual reminders, applying peer mentorship, and reorganizing ward spaces to reduce cross-contamination (8–10). These approaches are particularly relevant in resource-limited settings because they focus not only on technical infection control standards but also on feasibility, acceptability, staff behavior, workflow integration, and sustainability (10). Previous studies have shown that structured infection prevention and control interventions can reduce healthcare-associated infections and improve adherence to preventive practices (11). However, much of the available evidence comes from better-resourced settings or from interventions that require resources that may not be consistently available in low-resource hospitals. In addition, many studies have focused on short-term outcomes and have not sufficiently examined long-term sustainability, resource utilization, implementation barriers, or integration into routine ward operations (12). These gaps limit the transferability of existing evidence to hospitals operating under substantial resource constraints. Another important limitation in the literature is that many intervention studies rely on simple pre–post comparisons without adequately addressing secular trends, ward-level clustering, repeated measurements, or contextual factors that influence implementation. In resource-limited settings, changes in infection rates may be influenced by patient volume, staffing patterns, seasonal variation, supply availability, ward occupancy, and changes in the clinical case mix (13). Therefore, stronger implementation-focused evaluations are needed, using longitudinal outcome assessment, historical or comparison data where available, adjusted statistical models, and qualitative exploration of the implementation context. This study aimed to address these gaps by implementing and evaluating adaptive, context-specific infection prevention and control strategies in a resource-limited hospital setting. The intervention combined optimized equipment disinfection, low-cost staff training, peer mentorship, visual reminders, and spatial workflow reorganization. This study aimed to assess the effects of these strategies on hospital-acquired infection rates, evaluate healthcare worker adherence to infection prevention and control protocols, monitor resource utilization, and identify barriers and facilitators influencing implementation. By combining quantitative outcome evaluation with qualitative implementation analysis, this study provides a mixed-methods framework for assessing adaptive infection prevention and control interventions in resource-limited hospital settings.

## 2 Materials and methods

### 2.1 Study design and setting

This study was designed as a quasiexperimental, mixed-methods study. This study was conducted over a 12-months period from January 1, 2025, to December 31, 2025. Following a 1-month baseline assessment period in January 2025, the five intervention components were implemented simultaneously as an integrated multimodal bundle to maximize synergistic effects across the participating wards. This study was conducted within a major, 400-bed urban tertiary-level teaching facility located in Sana’a, Yemen, which serves as a central referral hub for a predominantly low-income population. The facility contains advanced specialty intensive care units, active surgical subspecialties, and extensive general inpatient blocks, meeting the national criteria for a tertiary-level referral institution. This study evaluated the effectiveness, feasibility, and sustainability of adaptive infection prevention and control strategies for reducing hospital-acquired infections and improving healthcare worker adherence to infection prevention protocols. Outcome assessments and surveillance data were collected over distinct, intensive 1-month periods at each designated time point: baseline (January 2025), 3 months (April 2025), 6 months (July 2025), and 12 months (December 2025), rather than through continuous surveillance throughout the entire year. Historical control data from the same wards during the preceding year were used to contextualize changes over time and support a two-period difference-in-differences comparison. Subgroup analyses compared intervention effects between intensive care unit wards and general inpatient wards.

### 2.2 Study population and participants

The study population included hospitalized patients and healthcare workers in the participating wards. Hospitalized patients were eligible for inclusion if they were admitted to one of the selected wards during the study period and remained hospitalized for at least 48 h. Patients were excluded from the primary hospital-acquired infection analysis if they had a documented infection at admission, were transferred from another hospital with an ongoing infection, or had incomplete infection surveillance records. The intervention cohort included 2,420 patient admissions contributing 13,980 patient-days across 12 wards, including 4 intensive care unit wards and 8 general inpatient wards. The historical control dataset included 2,310 admissions contributing 13,420 patient-days from comparable wards during the preceding year. Healthcare workers include physicians, nurses, cleaning staff, laboratory staff, and support personnel involved in patient care or infection prevention activities. A total of 126 healthcare workers were included in the structured adherence observations. A purposive sample of 38 healthcare workers participated in interviews or focus group discussions.

### 2.3 Intervention components

The intervention consisted of locally adapted infection prevention and control strategies designed to address resource constraints, workflow limitations, supply instability, and ward-specific infection risks. The intervention was implemented by the hospital infection prevention and control team in collaboration with ward supervisors and trained focal persons. The first component was optimized equipment disinfection. Existing cleaning and disinfection practices were reviewed, and locally available disinfectants were assessed for appropriate use in routine ward cleaning. Standardized disinfection protocols have been developed for reusable medical devices, shared equipment, bed rails, procedure surfaces, and high-touch areas. The second component was low-cost staff training. Healthcare workers received monthly training sessions lasting approximately 40–60 min. Training topics included hand hygiene, appropriate personal protective equipment use, safe handling of equipment, equipment disinfection, prevention of cross-contamination, and ward-specific infection risks. The third component was peer mentorship and ward-level reinforcement. Experienced staff and ward focal persons provided real-time reminders, demonstrated correct infection prevention practices, and reinforced adherence during routine clinical work. The fourth component was visual reminder support. Posters, quick-reference guides, color-coded signage, and floor markings were placed in relevant ward areas to support routine adherence and reduce reliance on memory. The fifth component was spatial workflow reorganization. Ward layouts and equipment stations were reorganized to improve separation between high-risk and low-risk patient zones and reduce unnecessary movement between contaminated and clean areas.

### 2.4 Outcomes

Hospital-acquired infections were defined according to the Centers for Disease Control and Prevention criteria (14, 15), specifically tracking central line-associated bloodstream infections (CLABSIs), catheter-associated urinary tract infections (CAUTIs), ventilator-associated pneumonia (VAP), and surgical site infections (SSIs) manifesting at least 48 h post-admission. The primary statistical modeling utilized the incidence density rate (per 1,000 patient-days) via Poisson or negative binomial regression with the logarithm of patient-days included as an exposure offset, whereas the cumulative incidence per 100 admissions was reported as a descriptive, complementary metric for institutional benchmarking. The secondary outcomes included adherence to infection prevention and control protocol components, resource utilization, and healthcare workers’ perceptions of implementation barriers and facilitators. Protocol adherence included observed compliance with hand hygiene, personal protective equipment use, and equipment disinfection. Resource utilization included disinfectant consumption, personal protective equipment consumption, infection prevention staff time, and training sessions. The qualitative outcomes included perceived barriers, facilitators, feasibility, acceptability, workload implications, peer mentorship, leadership support, and sustainability.

### 2.5 Data collection procedures

Quantitative data were collected at baseline and at 3, 6, and 12 months. The data included admissions, patient days, hospital-acquired infection events, ward type, length of stay, device exposure, and available patient risk indicators. Hospital-acquired infection surveillance was conducted via standardized infection control records and reviewed by trained infection prevention staff. Protocol adherence data were collected via structured observational checklists. Observations were conducted across different shifts and days of the week to reduce time-related observation bias. Each eligible opportunity was classified as compliant or noncompliant according to predefined checklist criteria. Hand hygiene opportunities were defined according to standard patient care. Personal protective equipment opportunities were defined according to clinical indications for protective equipment use. Equipment disinfection opportunities were defined as episodes requiring cleaning or disinfection before or after use. Resource utilization data were collected from hospital supply records, ward logs, and infection prevention and control activity reports. Disinfectant use was recorded in liters per month. The use of personal protective equipment was recorded as units per week and standardized every 100 patient-days. The duration of the infection prevention staff stay was recorded as hours per month. Training delivery was recorded as the number of sessions conducted per month. Qualitative data were collected through structured interviews and focus group discussions with healthcare workers. The interview guides explored perceived barriers to adherence, feasibility of the intervention, workload implications, acceptability of visual reminders, peer mentorship, perceived leadership support, and sustainability. Field notes and observation logs were used to support triangulation.

### 2.6 Sample size and precision considerations

Because the study was implemented under routine service conditions, all eligible admissions during the 12-months intervention period and the corresponding historical control period were included. The study therefore used a total-eligible-sample approach rather than prospective individual-level randomization. The intervention dataset included 2,420 admissions contributing 13,980 patient-days. The historical control dataset included 2,310 admissions contributing 13,420 patient-days. A precision-based sample adequacy assessment was conducted to determine whether the available sample was sufficient to estimate a clinically meaningful reduction in hospital-acquired infection incidence. Assuming a baseline infection incidence of approximately 15% and a minimum clinically important absolute reduction of 5 percentage points, approximately 650–750 admissions per comparison period were considered adequate to provide acceptable statistical precision. The available sample exceeded this threshold and was considered sufficient for the planned primary analysis. For adherence outcomes, all eligible directly observed infection prevention and control opportunities during the study period were included. Observation targets were set to provide stable adherence estimates across protocol components and evaluation periods.

### 2.7 Bias control and data quality assurance

Several procedures were used to reduce bias and improve data quality. Standardized hospital-acquired infection definitions were used, and infection events were classified according to the Centers for Disease Control and Prevention criteria (14, 15). Data collectors received training on case definitions, observation procedures, checklist completion, and data recording. To reduce observer variability, a subset of observations was independently assessed by two observers, and interrater agreement was calculated. To reduce the Hawthorne effect during adherence assessment, observations were conducted at varying times across shifts, and healthcare workers were not informed of the specific timing of the observation sessions. Historical control wards were selected on the basis of comparability with intervention wards. Comparability was assessed on the basis of ward type, admission volume, patient days, ward occupancy, baseline ward-level hospital-acquired infection rates, and available patient risk indicators. Patient days were used to standardize infection and resource utilization rates. Missing data were assessed before analysis. Records with missing infection outcome data were excluded from the primary analysis. Records with incomplete covariate data were retained where possible via available-case analysis. Sensitivity analyses were conducted to assess the robustness of the findings.

### 2.8 Statistical analysis

Quantitative analyses were conducted using ward-period, patient-level, and observation-level datasets. Descriptive statistics were used to summarize baseline characteristics, infection outcomes, adherence outcomes, and resource utilization. Owing to the relatively small absolute numbers of specific infection types when stratified across individual participating units, all healthcare-associated infections (HAIs) were analyzed collectively as a composite primary outcome to maintain adequate statistical power for the regression models. Categorical variables are summarized as frequencies and percentages. Continuous variables are summarized as the means with standard deviations or medians with interquartile ranges, depending on their distribution characteristics. The cumulative incidence of hospital-acquired infection was calculated as the number of hospital-acquired infection cases divided by the number of admissions and expressed as a percentage. The incidence density was calculated as the number of hospital-acquired infection cases per 1,000 patient-days. For infection rate modeling, Poisson regression was used with the logarithm of patient days included as an offset. Where overdispersion was detected, negative binomial regression was used instead. The results are reported as incidence rate ratios with 95% confidence intervals (CIs) and *p*-values. The models were adjusted for ward type and time point, with ward-level clustering accounting for the use of robust standard errors or random effects where appropriate. A two-period difference-in-differences analysis was conducted to compare the change in cumulative hospital-acquired infection incidence from baseline to 12 months in the intervention wards with the corresponding baseline-equivalent to follow-up equivalent change in the historical control wards. The difference-in-differences estimate was reported as an absolute percentage-point difference with 95% confidence intervals and *p*-values. The models were adjusted for ward type, patient days, and the baseline ward-level hospital-acquired infection rate. Protocol adherence outcomes were analyzed at the observation level. Each observed opportunity was treated as a binary outcome and classified as compliant or noncompliant. Mixed-effects logistic regression was used to estimate changes in adherence over time, with ward as a random effect and time point as a fixed effect. The results are reported as adjusted odds ratios with 95% confidence intervals. Resource utilization was summarized over time and standardized per 100 patient-days. Trends in disinfectant consumption, personal protective equipment use, infection prevention staff time, and training sessions were assessed via linear mixed-effects models, with the time point as a fixed effect and the ward as a random effect. Because no formal economic evaluation was conducted, the resource findings were interpreted as operational feasibility and resource utilization rather than cost-effectiveness. Prespecified subgroup analyses were conducted by ward type. The sensitivity analyses included models excluding the first implementation month, complete-case analyses, adjustments for device exposure, and ward-type-specific models. All the statistical tests were two-sided, and *p*-values less than 0.05 were considered statistically significant. Analyses were performed via SPSS.

### 2.9 Qualitative data analysis

Qualitative data from interviews, focus group discussions, field notes, and observation logs were analyzed via thematic analysis. The responses were transcribed and reviewed for familiarization. An initial coding framework was developed on the basis of the study objectives and emerging patterns in the data. The codes were grouped into themes representing barriers, facilitators, feasibility, acceptability, workload implications, peer mentorship, leadership support, and sustainability. To enhance credibility, a subset of transcripts was independently coded, and discrepancies were discussed until agreement was reached. The coding framework was refined and applied to the full qualitative dataset. Themes were summarized according to the number and percentage of participants mentioning each theme and were supported by illustrative quotations. Triangulation was performed across interviews, focus group discussions, field notes, and observation logs (Supplementary Appendix A). Qualitative findings were integrated with quantitative infection and adherence outcomes to provide a mixed-methods interpretation of implementation effectiveness.

### 2.10 Ethical considerations

The study was conducted in accordance with institutional ethical standards and principles of confidentiality. Ethical approval was obtained from the Hospitals Association Committee “Federation of Yemen Private Hospitals” (reference number 113–2025), and participants gave informed consent at the beginning of the surveys. All methods were carried out in accordance with the Declaration of Helsinki guidelines and regulations. Administrative approval was obtained from “A” Hospital before data collection. Patient-level data were extracted from routine surveillance and hospital records without identifying information. Because patient data were collected from routine anonymized records, individual patient consent was waived where applicable according to institutional approval procedures. Healthcare workers who participated in interviews or focus group discussions provided informed consent. Participation was voluntary and unrelated to workplace evaluation or employment status. All collected data were anonymized before analysis, stored securely, and reported only in aggregate form to protect participant confidentiality.

## 3 Results

### 3.1 Study population and baseline characteristics

Table 1 shows the baseline characteristics of the intervention cohort during the 12-months study period. A total of 2,420 patient admissions were included, contributing 13,980 patient-days across 12 wards, including 4 intensive care unit wards and 8 general inpatient wards. Compared with general wards, ICU wards had longer median lengths of stay, greater device exposure, and higher baseline HAI burdens. Overall, 126 healthcare workers were included in the adherence observations.

**TABLE 1:** Baseline characteristics of intervention wards and admitted patients.

Characteristic	ICU wards	General wards	Overall
Number of wards	4	8	12
Patient admissions, *n*	620	1,800	2,420
Patient-days, *n*	4,960	9,020	13,980
Mean age, years, mean ± SD	54.8 ± 16.2	47.3 ± 18.5	49.2 ± 18.1
Male sex, *n*/*N* (%)	352/620 (56.8)	986/1,800 (54.8)	1,338/2,420 (55.3)
Median length of stay, days, median (IQR)	7.5 (5–11)	4.2 (3–7)	5.1 (3–8)
Device exposure, *n*/*N* (%)	318/620 (51.3)	442/1,800 (24.6)	760/2,420 (31.4)
Baseline HAI cases, *n*/*N* (%)	35/159 (22.0)	59/454 (13.0)	94/613 (15.3)
Baseline HAI rate per 1,000 patient-days	27.1	16.8	19.7
Healthcare workers observed, *n*	42	84	126

HAI, hospital-acquired infection; ICU, intensive care unit; IQR, interquartile range; SD, standard deviation. Device exposure included urinary catheters, central venous catheters, mechanical ventilation, or other invasive devices. Baseline HAI denominators refer to admissions during the baseline assessment period only. Baseline HAI rates per 1,000 patient-days were calculated from patient-days from the baseline assessment period.

### 3.2 Hospital-acquired infection outcomes

Table 2 presents the changes in hospital-acquired infection outcomes across the 12-months intervention period. The overall cumulative HAI incidence decreased from 15.3% at baseline to 8.2% at 12 months, corresponding to an absolute reduction of 7.1 percentage points and a relative reduction of 46.4%. The overall incidence density decreased more modestly, from 19.7 to 17.1 infections per 1,000 patient-days. Both the cumulative incidence and incidence density of HAIs decreased in ICU wards, with the cumulative HAI incidence decreasing from 22.0% to 11.9% and the incidence density decreasing from 27.1 to 14.7 infections per 1,000 patient-days. The general ward showed a reduction in the cumulative HAI incidence from 13.0% to 7.6%; however, the incidence density increased from 16.8 to 20.7 infections per 1,000 patient-days, reflecting the lower number of patient-days during follow-up. In the adjusted negative binomial regression using log patient-days as an offset and accounting for ward-level clustering, the overall 12-months HAI rate was significantly lower than that at baseline, with an adjusted incidence rate ratio of 0.52 (95% CI: 0.35–0.77; *p* = 0.001).

**TABLE 2:** Changes in hospital-acquired infection outcomes across intervention wards.

Ward type	Time point	Admission *N*	Patient-days	HAI cases *N*	HAI cumulative incidence %	HAI rate per 1,000 patient-days	95% CI for rate	Adjusted IRR vs. baseline	95% CI	*P*-value
ICU	Baseline	159	1,292	35	22.0	27.1	19.1–37.6	Reference	–	–
	3 months	154	1,226	25	16.2	20.4	13.2–30.1	0.75	0.44–1.27	0.281
	6 months	156	1,218	19	12.2	15.6	9.4–24.3	0.58	0.32–1.05	0.071
	12 months	151	1,224	18	11.9	14.7	8.7–23.2	0.54	0.29–0.98	0.043
General wards	Baseline	454	3,512	59	13.0	16.8	12.8–21.6	Reference	–	–
	3 months	448	2,186	47	10.5	21.5	15.8–28.6	0.81	0.54–1.21	0.306
	6 months	450	1,678	35	7.8	20.9	14.5–29.1	0.61	0.39–0.95	0.028
	12 months	448	1,644	34	7.6	20.7	14.3–28.9	0.58	0.37–0.91	0.018
Overall	Baseline	613	4,804	94	15.3	19.7	15.9–24.0	Reference	–	–
	3 months	602	3,412	72	12.0	21.1	16.5–26.6	0.77	0.55–1.07	0.119
	6 months	606	2,896	54	8.9	18.6	14.0–24.3	0.60	0.41–0.86	0.006
	12 months	599	2,868	49	8.2	17.1	12.7–22.6	0.52	0.35–0.77	0.001

HAI, hospital-acquired infection; IRR, incidence rate ratio; CI, confidence interval. The incidence density was calculated per 1,000 patient-days. Adjusted IRRs were estimated via negative binomial regression with log patient-days as an offset and robust standard error clustered at the ward level. The models were adjusted for ward type and time point.

### 3.3 Comparison with historical control data

To examine whether the observed reduction in HAI incidence exceeded the expected secular variation, intervention wards were compared with historical control data from comparable wards during the preceding year. A multiperiod difference-in-differences longitudinal analysis across all intermediary intervals was precluded due to substantial gaps and missing monthly suballocations within the hospital’s historical paper records, which restricted the difference-in-differences matching strictly to baseline-equivalent and 12-months follow-up equivalent blocks. As shown in Table 3, the cumulative HAI incidence in the historical control group decreased modestly from 15.1% to 14.2%, corresponding to an absolute reduction of 0.9 percentage points. In contrast, the cumulative HAI incidence in the intervention wards decreased from 15.3% to 8.2%, corresponding to an absolute reduction of 7.1%. The adjusted difference-in-differences estimate revealed an additional 6.2-percentage-point reduction in cumulative HAI incidence in the intervention wards compared with historical controls. This estimate was statistically significant, supporting the likelihood that the observed improvement was associated with the adaptive IPC intervention rather than secular trends alone.

**TABLE 3:** Difference-in-differences comparison of cumulative HAI incidence between intervention wards and historical controls.

Group	Period	Admissions *N*	Patient-days	HAI cases *N*	Cumulative HAI incidence %	HAI rate per 1,000 patient-days	Absolute change, percentage points	Adjusted DiD estimate	95% CI	*P*-value
Historical control	Baseline-equivalent	585	3,420	88	15.1	25.7	Reference	Reference	–	–
	Follow-up equivalent	572	3,310	81	14.2	24.5	−0.9	Reference	–	–
Intervention wards	Baseline	613	4,804	94	15.3	19.7	Reference	Reference	–	–
	12 months	599	2,868	49	8.2	17.1	−7.1	−6.2	−9.8 to −2.6	0.001

DiD, difference-in-differences; CI, confidence interval. The DiD estimate represents the additional absolute percentage-point reduction in cumulative HAI incidence in intervention wards compared with historical controls. The model was adjusted for ward type, patient-days, and baseline ward-level HAI rate.

### 3.4 Protocol adherence outcomes

Adherence improved across all observed IPC protocol components following implementation of the adaptive strategies. As shown in Table 4, hand hygiene adherence increased from 55.1% at baseline to 83.0% at 12 months, corresponding to an absolute increase of 27.9%. PPE adherence increased from 60.2% to 80.1%, whereas equipment disinfection adherence increased from 50.1% to 76.1%. In the mixed-effects logistic regression accounting for clustering at the ward level, the odds of adherence at 12 months were significantly greater than those at baseline for all protocol components. The greatest 12-months improvement was observed for hand hygiene, followed by equipment disinfection and PPE use.

**TABLE 4:** Changes in adherence to IPC protocol components.

Protocol component	Time point	Observed opportunities *N*	Compliant actions *N*	Adherence %	95% CI	Absolute change vs. baseline, pp	Adjusted OR vs. baseline	95% CI	*P*-value
Hand hygiene	Baseline	1,020	562	55.1	52.0–58.1	Reference	Reference	–	–
	3 months	1,080	842	78.0	75.4–80.4	+22.9	2.86	2.35–3.48	<0.001
	6 months	1,120	953	85.1	82.9–87.1	+30.0	4.67	3.75–5.82	<0.001
	12 months	1,100	913	83.0	80.7–85.2	+27.9	4.21	3.39–5.23	<0.001
PPE use	Baseline	820	494	60.2	56.8–63.6	Reference	Reference	–	–
	3 months	850	638	75.1	72.1–78.0	+14.9	2.02	1.63–2.51	<0.001
	6 months	870	714	82.1	79.4–84.6	+21.9	3.05	2.40–3.88	<0.001
	12 months	860	689	80.1	77.3–82.7	+19.9	2.72	2.14–3.45	<0.001
Equipment disinfection	Baseline	760	381	50.1	46.5–53.7	Reference	Reference	–	–
	3 months	780	546	70.0	66.7–73.2	+19.9	2.31	1.88–2.84	<0.001
	6 months	810	624	77.0	73.9–79.8	+26.9	3.34	2.68–4.16	<0.001
	12 months	800	609	76.1	73.0–79.0	+26.0	3.18	2.55–3.97	<0.001

IPC, infection prevention and control; OR, odds ratio; CI, confidence interval; pp, percentage points. Adherence was calculated as compliant actions divided by observed opportunities. Adjusted ORs were estimated via mixed-effects logistic regression, with ward as a random effect and time point as a fixed effect.

### 3.5 Subgroup analysis by ward type

Table 5 presents 12-months changes in HAI outcomes and IPC adherence stratified by ward type. Both the ICU and general wards showed reductions in cumulative HAI incidence, with a greater absolute reduction observed in the ICU wards (−10.1 percentage points) than in the general wards (−5.4 percentage points). ICU wards also showed a reduction in HAI incidence density from 27.1 to 14.7 infections per 1,000 patient-days, whereas general wards showed an increase from 16.8 to 20.7 infections per 1,000 patient-days. The observed adherence to hand hygiene, PPE use, and equipment disinfection improved in both ward types. The general wards achieved slightly higher final adherence levels at 12 months, whereas the absolute improvement from baseline was slightly greater in the ICU wards. No statistically significant ward type-by-time interaction was observed for the primary HAI outcome, suggesting that the intervention effect on cumulative HAI incidence was broadly consistent across ward types.

**TABLE 5:** Ward-type subgroup analysis of 12-months changes in HAI outcomes and IPC adherence.

Outcome	ICU baseline	ICU 12 months	ICU change	General baseline	General 12 months	General change	Interaction *P*-value
HAI cumulative incidence, %	22.0	11.9	−10.1 pp	13.0	7.6	−5.4 pp	0.184
HAI rate per 1,000 patient-days	27.1	14.7	−45.8%	16.8	20.7	+23.2%	0.096
Hand hygiene adherence, %	52.4	81.6	+29.2 pp	56.2	83.7	+27.5 pp	0.611
PPE adherence, %	58.1	78.4	+20.3 pp	61.1	80.9	+19.8 pp	0.742
Equipment disinfection adherence, %	48.3	74.8	+26.5 pp	51.0	76.8	+25.8 pp	0.801

HAI, hospital-acquired infection; ICU, intensive care unit; IPC, infection prevention and control; PPE, personal protective equipment; pp, percentage points. Changes in cumulative incidence and adherence are expressed as percentage-point differences. Changes in the HAI rate per 1,000 patient-days are expressed as relative percentage changes.

### 3.6 Resource utilization

Table 6 summarizes the changes in resource utilization during the intervention period. Disinfectant consumption increased from 12.1 to 17.6 liters per month, whereas PPE use increased from 201 to 229 units per week. The IPC staff time increased from 42.5 to 56.8 h per month, reflecting mainly training, supervision, and observation activities. Training sessions were introduced during the intervention period and were maintained at approximately 3.6–4.0 sessions per month after implementation. After standardization by patient-days, resource use remained within operationally manageable levels. Because no formal economic evaluation was conducted, these findings were interpreted as evidence of implementation feasibility and manageable resource requirements rather than cost effectiveness.

**TABLE 6:** Resource utilization before and after implementation of adaptive IPC strategies.

Resource	Unit	Baseline mean ± SD	3 months mean ± SD	6 months mean ± SD	12 months mean ± SD	Relative change baseline to 12 months	Standardized 12-months rate	*p* for trend
Disinfectant use	Liters/month	12.1 ± 1.8	16.2 ± 2.1	17.1 ± 2.4	17.6 ± 2.3	+45.5%	0.61 L/100 patient-days	0.003
PPE use	Units/week	201 ± 24	221 ± 28	226 ± 31	229 ± 29	+13.9%	31.9 units/100 patient-days	0.021
IPC staff time	Hours/month	42.5 ± 6.7	53.2 ± 7.1	55.9 ± 7.4	56.8 ± 7.8	+33.6%	1.98 h/100 patient-days	0.008
Training sessions delivered	Sessions/month	0.0 ± 0.0	3.8 ± 0.8	4.0 ± 0.7	3.6 ± 0.6	Not applicable	0.13 sessions/100 patient-days	<0.001

IPC, infection prevention and control; PPE, personal protective equipment; SD, standard deviation. Trends were assessed via linear mixed-effects models, with the time point as a fixed effect and the ward as a random effect. Standardized 12-months rates were calculated per 100 patient-days to account for differences in patient volume over time. Resource utilization outcomes were interpreted as operational indicators; no formal cost-effectiveness or economic evaluation was conducted.

### 3.7 Qualitative findings: barriers and facilitators

Thirty-eight healthcare workers participated in the qualitative component, including 21 nurses, 8 physicians, 5 cleaning staff, and 4 support staff. Thematic analysis identified three frequently reported barriers and three facilitators influencing implementation. Barriers were related mainly to operational pressures and resource constraints, including high patient loads, occasional shortages of disinfectants and PPE, and workflow interruptions during peak hours. Facilitators were related mainly to practical behavioral support and ward-level reinforcement, including visual reminders, peer mentorship, and leadership engagement. Overall, the participants described the adaptive IPC intervention as feasible and acceptable when it was supported by visible reminders, peer reinforcement, and managerial follow-up. The detailed themes, participant frequencies, illustrative quotations, and interpretations are presented in Table 7.

**TABLE 7:** Qualitative themes related to barriers and facilitators of implementation.

Theme type	Theme	Participants mentioning theme, *n*/*N* (%)	Illustrative quote	Interpretation
Barrier	High patient load	25/38 (65.8)	“During peak hours, it becomes difficult to follow every step, especially when many patients need care at the same time.”	Workload pressure reduced consistent adherence during busy shifts.
	Occasional shortages of disinfectants and PPE	15/38 (39.5)	“Sometimes the protocol is clear, but the supplies are not always available when needed.”	Supply instability limited full implementation of IPC practices.
	Workflow interruptions during peak hours	13/38 (34.2)	“The process works better when the ward is organized, but interruptions make it harder to complete disinfection immediately.”	Workflow disruption affected timely completion of IPC steps.
Facilitator	Visual reminders	31/38 (81.6)	“The posters and signs helped us remember the correct steps without needing to ask each time.”	Visual cues supported routine adherence and reduced reliance on memory.
	Peer mentorship	27/38 (71.1)	“When senior staff reminded us during the shift, it became easier to follow the protocol.”	Peer support reinforced behavior change during routine work.
	Leadership engagement	25/38 (65.8)	“When the unit manager followed up, everyone became more committed.”	Leadership support improved accountability and sustainability.

The percentages are based on the 38 healthcare workers who participated in interviews or focus group discussions. Themes were derived via thematic analysis and triangulated with observation logs. IPC, infection prevention and control; PPE, personal protective equipment.

### 3.8 Sensitivity analyses

Sensitivity analyses were conducted to evaluate the primary HAI outcome (detailed model specifications for the sensitivity analyses are presented in Supplementary Table 1). The reduction in HAI rates at 12 months remained statistically significant after excluding the first month following intervention implementation to account for the transition period. The complete-case analysis produced effect estimates similar to those of the primary model. Additional adjustment for device exposure did not materially change the direction or magnitude of the intervention effect. Ward type-specific models also revealed significant reductions in both ICUs and general wards, supporting the robustness of the primary findings.

## 4 Discussion

This quasiexperimental implementation-focused study demonstrated that adaptive infection prevention and control strategies were associated with substantial reductions in hospital-acquired infection rates over a 12-months period and significant improvements in healthcare worker adherence to IPC protocols in a resource-limited hospital setting. Over the 12-months follow-up period, the overall cumulative incidence of HAI decreased from 15.3% at baseline to 8.2% at 12 months, corresponding to an absolute reduction of 7.1 percentage points and a relative reduction of approximately 46.4%. After adjustment for patient days, ward type, time point, and ward-level clustering, the 12-months HAI incidence rate remained significantly lower than that at baseline, with an adjusted incidence rate ratio of 0.52, indicating an approximately 48% adjusted reduction in HAI incidence. Overall, the HAI cumulative incidence decreased, and adjusted rate models revealed a statistically significant reduction in HAI rates over time. However, the incidence density increased in general wards from 16.8 to 20.7 infections per 1,000 patient-days, and this subgroup finding should be interpreted cautiously because patient-days decreased during follow-up. Importantly, the observed postintervention increase in HAI incidence density within general units may reflect a pronounced “surveillance effect.” The implementation of structured peer mentorship, regular training, and mandatory checklist recording likely improved the clinical detection and institutional reporting of baseline nosocomial conditions that previously escaped documentation owing to suboptimal surveillance coverage. The ICU wards had the highest baseline infection burden, with a marked decline from 22.0% to 11.9%, whereas the burden in the general wards decreased from 13.0% to 7.6%. This pattern is clinically plausible because ICU wards usually have higher baseline infection risks due to invasive device use, longer lengths of stay, more severely ill patients, and greater exposure to high-risk procedures (16). The observed improvements suggest that adaptive interventions may be particularly useful in high-risk wards when they are integrated into routine clinical workflows and tailored to local resource constraints. A key strength of the present study is that it did not rely only on a simple pre–post comparison. The inclusion of historical control data and the application of difference-in-differences analysis strengthened causal interpretation by helping to separate intervention-related effects from secular trends. While historical control wards showed only a modest reduction in HAI incidence, the intervention wards showed a substantially greater decline. The adjusted difference-in-differences estimate indicated an additional absolute reduction of 6.2 percentage points in HAI incidence in the intervention wards compared with historical controls. This finding supports the interpretation that the observed reduction was likely associated with the adaptive IPC intervention rather than being explained solely by background variation or routine temporal changes. The improvement in protocol adherence provides an important explanatory pathway for the reduction in HAI outcomes. Hand hygiene adherence increased from 55.1% to 83.0%, PPE adherence increased from 60.2% to 80.1%, and equipment disinfection adherence increased from 50.1% to 76.1%. According to adjusted mixed-effects logistic regression, the odds of adherence at 12 months were significantly greater than those at baseline for all IPC components. The strongest improvement was observed for hand hygiene, followed by equipment disinfection and PPE use. These findings indicate that behavioral and workflow-based interventions, when delivered repeatedly and reinforced through visual reminders and peer support, can produce measurable and sustained changes in clinical practice. The resource utilization findings are also important for interpreting feasibility in a low-resource context. Disinfectant consumption, PPE use, and IPC staff time increased during the intervention period, but the increases were modest after standardization by patient-days. Therefore, the results should be interpreted as evidence of resource-efficient implementation rather than formal cost-effectiveness. A full economic evaluation was not conducted in this study; therefore, claims about cost-effectiveness or cost savings should be avoided. Nevertheless, the findings suggest that meaningful improvements in IPC adherence and HAI outcomes can be achieved without excessive additional resource demands, which is particularly relevant for hospitals operating under supply and budget constraints. The qualitative findings further clarify why the intervention was feasible and sustainable. Staff identified high patient loads, occasional shortages of disinfectants and PPE, and workflow interruptions during peak hours as the main implementation barriers. These barriers are highly relevant in resource-limited hospitals, where infection control practices are often affected not only by knowledge gaps but also by workload, supply instability, space constraints, and competing clinical priorities. Moreover, visual reminders, peer mentorship, and leadership engagement were consistently identified as facilitators. These findings suggest that successful IPC implementation depends on both technical measures and organizational support. These findings are consistent with prior research on implementation-focused IPC interventions in resource-limited settings (17). For example, Latif et al. reported that structured infection control bundles substantially reduced HAI incidence across hospitals in Pakistan (11). Similarly, Chakma et al. reported that behavioral interventions, including repeated training and visual cues, improved hand hygiene compliance from 66.0% to 88.3% (18). The present study extends this evidence by combining HAI surveillance, direct adherence observation, resource utilization monitoring, historical control comparison, and qualitative implementation analysis within a single adaptive IPC framework. The barriers identified in this study also align with previous evidence. Afework et al. reported that high workload and time pressure remain persistent challenges to IPC adherence, especially in ICUs, emergency departments, and busy inpatient wards (19). Similarly, Von Auer et al. highlighted the effectiveness of visual reminders and behavioral nudges, including posters, signs, and campaigns, in promoting adherence to infection control practices (20). The present findings support these observations and suggest that visual cues may be especially effective when combined with peer mentorship, leadership follow-up, and workflow reorganization. Another important contribution of this study is its mixed-methods interpretation of implementation effectiveness. The quantitative findings revealed significant reductions in HAI rates and significant improvements in protocol adherence, whereas the qualitative findings explained the contextual mechanisms that supported or limited implementation. This integration is particularly important in resource-limited settings, where intervention success depends not only on whether the technical protocol is evidence-based but also on whether it is practical, acceptable, affordable, and compatible with everyday ward operations (21). Overall, this study suggests that adaptive, context-specific IPC strategies can be implemented successfully in resource-limited hospitals and may lead to sustained improvements in patient safety. The findings support the value of combining low-cost staff training, optimized equipment disinfection, visual reminders, spatial reorganization, peer mentorship, and leadership engagement. However, the findings should be interpreted in light of the quasiexperimental design, the single-hospital setting, and the use of historical rather than concurrent randomized controls.

### 4.1 Implications for practice

The findings have several practical implications for hospitals in resource-limited settings. First, IPC improvement does not necessarily require expensive technologies or large-scale infrastructure changes. When standardized and embedded into routine workflows, locally adapted strategies can produce meaningful improvements in infection control outcomes. Second, direct observation of adherence should be incorporated into routine IPC monitoring because infection outcomes alone may not identify which behavioral or procedural components require improvement. Third, visual reminders and peer mentorship appear to be practical tools for sustaining adherence over time. Posters, quick-reference guides, color-coded signage, and floor markings can reduce reliance on memory and support consistent practice during busy shifts. Peer mentorship can reinforce correct behavior in real time without requiring continuous formal supervision. Fourth, leadership engagement is essential. Unit managers and IPC leaders play a central role in maintaining accountability, ensuring supply availability, supporting workflow adaptations, and reinforcing staff commitment. Finally, resource utilization should be monitored alongside clinical outcomes. In this study, improved adherence was achieved, with only modest increases in disinfectant use, PPE consumption, and IPC staff time after standardization by patient-days. This suggests that adaptive IPC strategies can be operationally feasible in constrained settings. However, formal cost-effectiveness claims require dedicated economic evaluation and should not be assumed from resource utilization data alone.

### 4.2 Limitations

This study has several limitations. First, the study was conducted in a single tertiary hospital in Yemen, which may limit the generalizability of the findings to other hospitals or healthcare systems. Resource availability, staffing levels, patient volume, ward design, and IPC culture may differ across settings. Therefore, the intervention framework should be adapted and re-evaluated before implementation in other contexts. Second, historical controls were used instead of concurrent randomized controls. Although historical comparison helped reduce the limitations of a simple pre–post design, it may not fully account for temporal changes, seasonal variation, or unmeasured differences between periods. Future studies should consider multicenter controlled designs or cluster-randomized implementation trials where feasible. Third, adherence outcomes were based on direct observation, which may be affected by the Hawthorne effect. Although observations were conducted across different shifts and times to reduce this risk, healthcare workers may have modified their behavior when they were aware of being observed. The use of unobtrusive monitoring or automated adherence tracking could provide additional validation in future studies. Fourth, outcome data collection was restricted to specific intervals (3, 6, and 12 months), and a 9-months assessment was omitted because temporary infection control staffing shortages and acute operational resource constraints disrupted active surveillance activities during that specific quarter. Fifth, the exclusion of patient records with missing primary infection outcome data could introduce selection bias, although these missing records constituted less than 3% of the total eligible admissions, and sensitivity analyses demonstrated that the effect estimates remained robust. Sixth, the 12-months study timeframe prevents a definitive assessment of long-term operational sustainability. True institutional sustainability can be verified only by evaluating infection rates 2–3 years post-intervention to confirm whether protocol adherence and behavioral adaptations are permanently integrated into routine hospital operations after active monitoring ends. Seventh, the study did not conduct a formal cost-effectiveness analysis. Resource utilization was measured and standardized per patient-day, but direct costs, indirect costs, cost per HAI prevented, and potential savings from reduced infections were not fully estimated. Therefore, the findings should be interpreted as evidence of operational feasibility and resource-efficient implementation, not definitive economic cost-effectiveness. Eighth, the qualitative findings were based on staff interviews and focus groups and may be influenced by social desirability bias. Staff may have overreported positive perceptions of the intervention or underreported barriers related to leadership, workload, or resource constraints. Triangulation with observation logs helps strengthen credibility, but future studies could include anonymous surveys or external qualitative evaluations.

### 4.3 Future research directions

Future research should evaluate adaptive IPC strategies via multicenter study designs across diverse resource-limited healthcare settings. Cluster-randomized trials, stepped-wedge designs, or controlled interrupted time series studies would provide stronger evidence regarding the effectiveness and generalizability of the findings. Such studies should include larger numbers of hospitals, wards, patients, and healthcare workers to assess whether the intervention remains effective across different organizational contexts. Long-term follow-up beyond 1 year is also needed to determine whether improvements in HAI rates and protocol adherence are sustained after the initial implementation period. Sustainability should be assessed not only through infection outcomes but also through staff turnover, training continuity, supply availability, leadership engagement, and the integration of IPC practices into routine hospital governance. Future studies should also include formal economic evaluations. Cost-effectiveness, cost-benefit, or cost-utility analyses could estimate the cost per HAI prevented, the financial savings from reduced infection burden, and the affordability of scaling adaptive IPC interventions across low-resource hospitals. Such evidence would be particularly valuable for policymakers and hospital administrators. Further research should explore the role of digital or automated reminder systems in supporting IPC adherence. Mobile alerts, electronic dashboards, QR-based checklists, and automated hand hygiene monitoring systems may offer scalable options, although their feasibility in resource-limited settings requires careful evaluation. Hybrid models that combine low-cost visual reminders with simple digital monitoring tools may be especially promising. Finally, future qualitative research should examine how organizational culture, leadership behavior, staff motivation, and supply chain reliability influence IPC implementation. Understanding these contextual determinants will help refine adaptive intervention models and improve their transferability across different healthcare environments.

## 5 Conclusion

Adaptive, context-specific infection prevention and control strategies are highly effective and operationally feasible for reducing healthcare-associated infection rates and increasing compliance in resource-limited hospital settings. By deploying low-cost training, prominent visual reminders, peer mentorship, and spatial reorganization, meaningful behavioral and clinical improvements can be achieved and maintained over 12 months with manageable increases in resource use. These findings offer a practical, scalable public health template for improving patient safety in constrained healthcare environments globally. Future multicenter trials are needed to confirm the long-term sustainability and economic scalability of these interventions.

## Data Availability

The original contributions presented in this study are included in this article/Supplementary material, further inquiries can be directed to the corresponding author.
