# Supplementary material for: Implementing adaptive strategies for infection prevention and control in resource-limited settings
Source: Front Med (Lausanne). 2026 Jul 7;13:1883213. doi: 10.3389/fmed.2026.1883213 (PMC13386225; doi:10.3389/fmed.2026.1883213)
Supplement: Supplementary file 1 [file Table_1.docx]

**Supplementary Material: Appendix A**

**Infection Prevention and Control (IPC) Observational Checklist**

**Purpose:** This structured observational tool is designed for real-time, unobtrusive monitoring of healthcare worker (HCW) compliance with Hand Hygiene (HH), Personal Protective Equipment (PPE) use, and equipment disinfection protocols across variable shifts and ward types.

**1. General Metadata**

- **Date of Observation:** [DD/MM/YYYY]
- **Observation Shift:** Morning | Afternoon | Night
- **Ward Type:** Intensive Care Unit (ICU) | General Inpatient Ward
- **Ward/Unit Name:** [e.g., Male Medical, Surgical ICU] ………………
- **HCW Category:** Physician | Nurse | Cleaning Staff | Other Support Staff
- **Observer ID:** ………………..

**2. Section A: Hand Hygiene Compliance Monitoring**

(Based on the WHO 5 Moments for Hand Hygiene, adapted for rapid clinical auditing)

| **Opportunity ID** | **Indication / Moment** | **Care Activity Description** | **Compliant (Yes / No)** | **Action Taken (Alcohol Rub / {Soap & Water})** |
| --- | --- | --- | --- | --- |
| **01** | **Moment 1:** Before touching a patient | E.g., Prior to shaking hands, measuring vitals. | Y / N | Rub / Wash |
| **02** | **Moment 2:** Before clean/aseptic procedure | E.g., Insertion of peripheral IV, wound dressing. | Y / N | Rub / Wash |
| **03** | **Moment 3:** After body fluid exposure risk | E.g., Emptying urinary catheter bag, blood draw. | Y / N | Rub / Wash |
| **04** | **Moment 4:** After touching a patient | E.g., After physical examination, moving patient. | Y / N | Rub / Wash |
| **05** | **Moment 5:** After touching patient surroundings | E.g., Adjusting bed rails, clearing bedside table. | Y / N | Rub / Wash |

**Note: To be marked as Compliant (Yes), the HCW must perform the hand friction rub for at least 20–30 seconds, or hand wash with soap and water for 40–60 seconds, ensuring all surfaces of the hands are covered.*

**3. Section B: Personal Protective Equipment (PPE) Compliance**

(Evaluated based on clinical indication and correct technique during the care episode)

| **PPE Item** | **Indication for Use Verified?** | **Correct Donning Sequence & Fit?** | **Correct Doffing Sequence (No Self-Contamination)?** | **Disposal in Designated Infectious Waste Container?** | **Overall Item Compliance** |
| --- | --- | --- | --- | --- | --- |
| **Gloves** | Yes / No | Yes / No | Yes / No | Yes / No | Compliant / Non-Compliant |
| **Gown / Apron** | Yes / No | Yes / No | Yes / No | Yes / No | Compliant / Non-Compliant |
| **Medical Mask** | Yes / No | Yes / No | Yes / No | Yes / No | Compliant / Non-Compliant |
| **Eye Protection** | Yes / No | Yes / No | Yes / No | Yes / No | Compliant / Non-Compliant |

- **Total PPE Episode Evaluation:** **Compliant** (All indicated items worn and discarded correctly) | **Non-Compliant** (Any step failed)

**4. Section C: Shared Medical Equipment Disinfection Compliance**

(To be filled during episodes involving non-disposable medical devices such as stethoscopes, BP cuffs, thermometers, glucometers, or bed rails)

| **Equipment Type** | **Opportunity Context** | **Verified Use of Approved Local Disinfectant Solution?** | **Proper Contact Time / Friction Applied?** | **Overall Compliance** |
| --- | --- | --- | --- | --- |
| [e.g., Stethoscope] | Before Patient Use / After Patient Use | Yes / No | Yes / No | Y / N |
| [e.g., BP Cuff] | Before Patient Use / After Patient Use | Yes / No | Yes / No | Y / N |
| [e.g., Bed Rails] | Routine Shift Cleaning / Post-Discharge | Yes / No | Yes / No | Y / N |

**5. Summary Metrics for the Observation Session**

- **Total Hand Hygiene Opportunities Observed:** [**] | Total Compliant: [**] rightarrow **Session Adherence:** ……%
- **Total PPE Use Opportunities Observed:** [**] | Total Compliant: [**] rightarrow
- **Session Adherence:** …..%
- **Total Equipment Disinfection Opportunities Observed:** [**] | Total Compliant: [**] rightarrow **Session Adherence:** …..%

**Observer Qualitative Notes (Optional):** (e.g., supply shortages observed during shift, high patient volume constraints affecting workflow)
