# Supplementary material for: Implementing adaptive strategies for infection prevention and control in resource-limited settings
Source: Front Med (Lausanne). 2026 Jul 7;13:1883213. doi: 10.3389/fmed.2026.1883213 (PMC13386225; doi:10.3389/fmed.2026.1883213)
Supplement: Supplementary file 2 [file Table_2.docx]

| Table S1. Sensitivity analyses of the adjusted intervention effect on HAI rates | | | | |
| --- | --- | --- | --- | --- |
| **Analysis model** | **Adjusted IRR for 12 months vs baseline** | **95% CI** | **p value** | **Interpretation** |
| Primary model | 0.52 | 0.35–0.77 | 0.001 | Significant reduction in HAI rate |
| Excluding first implementation month | 0.55 | 0.37–0.81 | 0.003 | Effect remained significant after transition period exclusion |
| Complete-case analysis only | 0.54 | 0.36–0.80 | 0.002 | Findings robust to missing data exclusion |
| Adjusted for device exposure | 0.57 | 0.39–0.84 | 0.005 | Effect persisted after adjustment for patient risk |
| ICU-only model | 0.54 | 0.29–0.98 | 0.043 | Significant reduction in ICU wards |
| General-ward-only model | 0.58 | 0.37–0.91 | 0.018 | Significant reduction in general wards |
| **Note. IRR = incidence rate ratio; CI = confidence interval. All models used patient days as the exposure denominator, with log patient days included as an offset.** | | | | |
